# Cancer combination therapies by angiogenesis inhibitors; a comprehensive review

**DOI:** 10.1186/s12964-022-00838-y

**Published:** 2022-04-07

**Authors:** Mohammad Javed Ansari, Dmitry Bokov, Alexander Markov, Abduladheem Turki Jalil, Mohammed Nader Shalaby, Wanich Suksatan, Supat Chupradit, Hasan S. AL-Ghamdi, Navid Shomali, Amir Zamani, Ali Mohammadi, Mehdi Dadashpour

**Affiliations:** 1 Department of Pharmaceutics, College of Pharmacy, Prince Sattam Bin Abdulaziz University, Al-Kharj, Kingdom of Saudi Arabia; 2 Institute of Pharmacy, Sechenov First Moscow State Medical University, 8 Trubetskaya St., bldg. 2, Moscow, 119991 Russian Federation; 3 Laboratory of Food Chemistry, Federal Research Center of Nutrition, Biotechnology and Food Safety, 2/14 Ustyinsky pr., Moscow, 109240 Russian Federation; 4 Tyumen State Medical University, Tyumen, Russian Federation; 5 Industrial University, Tyumen, Russian Federation; 6 Faculty of Biology and Ecology, Yanka Kupala State University of Grodno, 230023 Grodno, Belarus; 7 College of Technical Engineering, The Islamic University, Najaf, Iraq; 8 Department of Dentistry, Kut University College, Kut, Wasit 52001 Iraq; 9 Biological Sciences and Sports Health Department, Faculty of Physical Education, Suez Canal University, Ismailia, Egypt; 10 Faculty of Nursing, HRH Princess Chulabhorn College of Medical Science, Chulabhorn Royal Academy, Bangkok, Thailand; 11 Department of Occupational Therapy, Faculty of Associated Medical Sciences, Chiang Mai University, Chiang Mai, 50200 Thailand; 12 Internal Medicine Department, Division of Dermatology, Albaha University, Al Bahah, Kingdom of Saudi Arabia; 13 Immunology Research Center (IRC), Tabriz University of Medical Sciences, Tabriz, Iran; 14 Shiraz Transplant Center, Abu Ali Sina Hospital, Shiraz University of Medical Sciences, Shiraz, Iran; 15 Department of Neurology, Imam Khomeini Hospital, Urmia University of Medical Sciences, Urmia, Iran; 16 Department of Medical Biotechnology, Faculty of Medicine, Semnan University of Medical Sciences, Semnan, Iran

**Keywords:** Angiogenesis, Tumor, Anti-angiogenic agents, Combination therapy, Resistance

## Abstract

Abnormal vasculature is one of the most conspicuous traits of tumor tissue, largely contributing to tumor immune evasion. The deregulation mainly arises from the potentiated pro-angiogenic factors secretion and can also target immune cells' biological events, such as migration and activation. Owing to this fact, angiogenesis blockade therapy was established to fight cancer by eliminating the nutrient and oxygen supply to the malignant cells by impairing the vascular network. Given the dominant role of vascular-endothelium growth factor (VEGF) in the angiogenesis process, the well-known anti-angiogenic agents mainly depend on the targeting of its actions. However, cancer cells mainly show resistance to anti-angiogenic agents by several mechanisms, and also potentiated local invasiveness and also distant metastasis have been observed following their administration. Herein, we will focus on clinical developments of angiogenesis blockade therapy, more particular, in combination with other conventional treatments, such as immunotherapy, chemoradiotherapy, targeted therapy, and also cancer vaccines.

**Video abstract**

**Supplementary Information:**

The online version contains supplementary material available at 10.1186/s12964-022-00838-y.

## Introduction

Angiogenesis is a critical process that is needed for many physiological and pathological activities [1]. Angiogenesis is a heavily controlled process under physiological circumstances. It usually happens throughout embryonic development, wound repair, and the menstrual cycle [2]. Under physiological circumstances, angiogenesis relies on the equilibrium of positive and negative angiogenic modulators within the vascular microenvironment and necessitates the contribution of diverse molecules, such as pro-angiogenic factors, extracellular matrix (ECM) proteins, adhesion receptors, and also proteolytic enzymes [3]. Pathological diseases including psoriasis, diabetic retinopathy, as well as cancer exhibit unregulated angiogenesis. Angiogenesis is necessary during tumor development for appropriate feeding and elimination of metabolic waste products from tumor regions [4]. In reality, tumor development and metastasis are dependent on angiogenesis as well as lymphangiogenesis, which are initiated by chemical impulses from cancer cells in a fast-growing phase [5, 6]. Muthukkaruppan and colleagues previously investigated the dynamics of cancer cells injected into various areas of the same organs [7]. One part was the iris, which had blood circulation, and the other was the anterior chamber, which did not [7]. Cancer cells lacking blood circulation expanded 1–2 mm^3^ in diameter and afterward halted, but when put in a location where angiogenesis was feasible, they expanded to more than 2 mm^3^. Given that tumors become necrotic or even apoptotic in the absence of a circulatory supply [8], it has strongly been validated that angiogenesis is a critical component in cancer development.

**Fig. 1:**
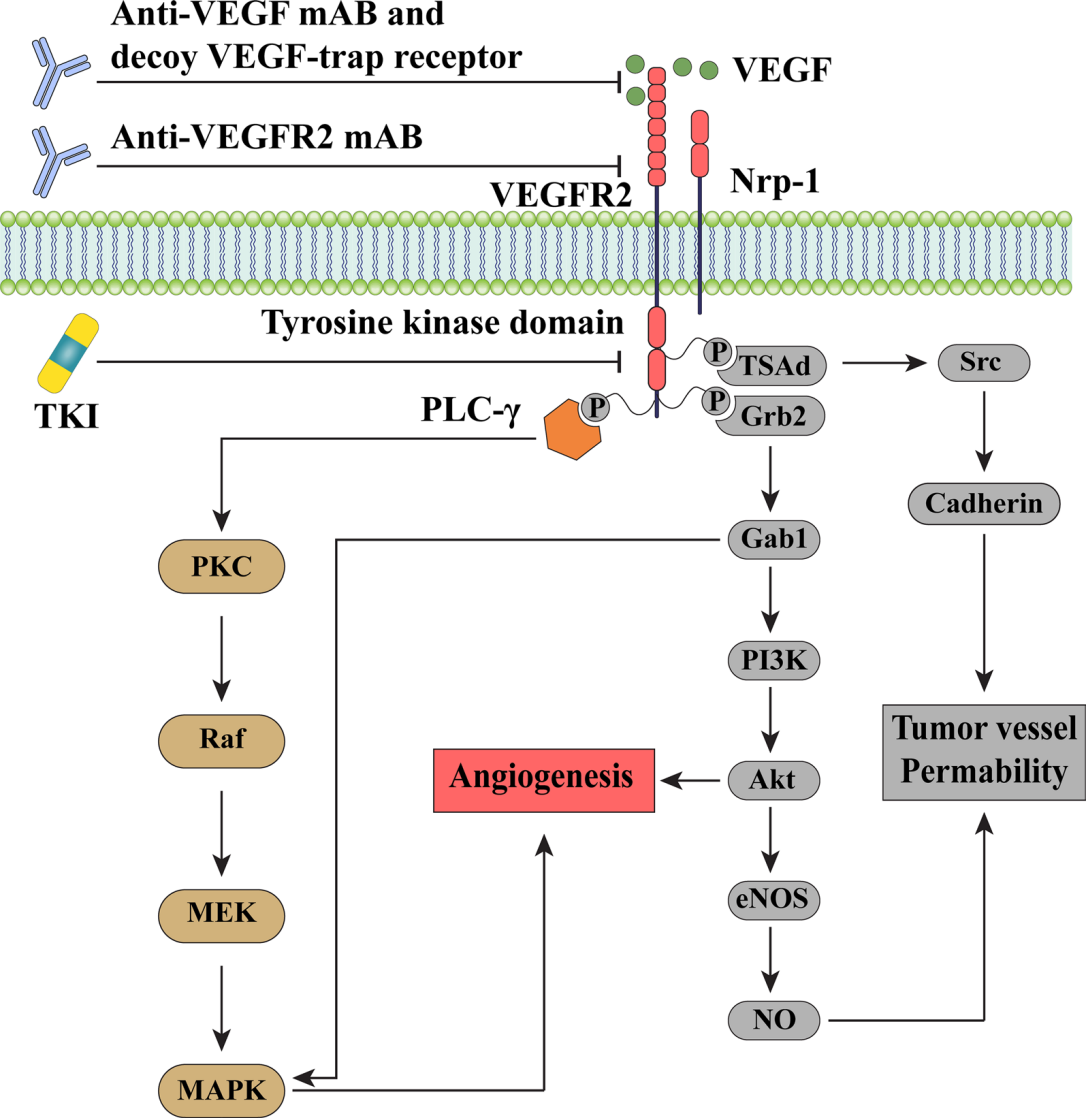
The central role of VEGF in tumor angiogenesis. The VEGF induces angiogenesis in tumor cells following interaction with responding receptor, VEGFR2, on tumor cells and subsequently by activating various signaling axes. In contrast, targeting VEGF/VEGFR2 using TKI or monoclonal anti-body could be applied to fence tumor angiogenesis and development

Tumors differ significantly in the patterns and characteristics of the angiogenic vascular system, as well as their sensitivity to anti-angiogenic treatment [9]. Cancer cells control the angiogenic programming of neoplastic tissues through collaboration with a range of tumor-associated stromal cells as well as their bioactive products, which include cytokines and growth hormones, the extracellular matrix, as well as secreted microvesicles [10]. Apart from cancer immunotherapy or other pioneering approaches such as chemotherapy and radiotherapy, which have resulted in a significant advance in cancer treatment [11, 12], another potential treatment approach is anti-angiogenesis, which aims to impair the vasculature and deprive the tumor of oxygen and nutrition [13]. This is accomplished mostly by targeting the pro-angiogenic factors-induced signaling pathway, which is prominent in the tumor microenvironment under hypoxic conditions [14]. Pro-angiogenic factors are classified into two main subgroups: (1) classical, including vascular endothelial growth factor (VEGF), fibroblast growth factor-2 (FGF-2), platelet-derived growth factor (PDGF), platelet-derived endothelial cell growth factor/thymidine phosphorylase (PD-ECGF/TP), angiopoietins (Ang), hepatocyte growth factor (HGF), insulin-like growth factors (IGFs), tumor necrosis factor (TNF), interleukin-6 (IL-6); (2) non-classical, including stem cell factor (SCF), tryptase and also chymase [15]. VEGF family members are the regulator of angiogenesis both under normal circumstances and in a disease condition. This family consists of VEGF-A, VEGF-B, VEGF-C, VEGF-D, VEGF-E, and placenta growth factor (PlGF), which binds with divergent affinities and specificities to tyrosine kinase receptors (VEGFR) 1,-2, and -3 [16, 17]. The interfaces between VEGF-A and VEGFR 2 exceed angiogenesis, while VEGF-C and D preferentially make connections with VEGFR-3 [18]. The improved expression of VEGF inspires tumourigenesis by potentiating the epithelial-mesenchymal transition (EMT) activation. In addition to VEGF receptor tyrosine kinases, the neuropilins (NRPs), potent co-receptors for class 3 semaphorins, are crucial for exerting the impacts of VEGF on cancer cells as a result of their capability to affect the activities of growth factor receptors and integrins [19]. VEGF/NRP axis adjusts the expression and action of important biological molecules, such as Rho family guanosine triphosphatases (GTPases) and transcription factors in malignant cells [20]. Respecting the pivotal role of the VEGF/VEGFR signaling axis in cancer angiogenesis, several anti-angiogenic medicines have been authorized for various types of cancer, such as anti-VEGF antibodies, anti-VEGFR antibodies, and VEGFR tyrosine kinase inhibitors (TKIs) (Fig. 1) [14, 21]. Meanwhile, multitargeted small-molecule TKI can target multiple receptor sites simultaneously. The main targets included vascular endothelial growth factor receptor (VEGFR), platelet-derived growth factor receptor (PDGFR), fibroblast growth factor receptor (FGFR), c-Kit, and c-Met. Anti-angiogenic TKIs block the kinase activity of receptors and transduction of downstream signals involved in the proliferation, migration, and survival [22]. However, monotherapy with an anti-angiogenic drug has shown minimal therapeutic advantages for most cancer patients [23]. Thereby, it has been suggested and also evidenced that combining anti-angiogenic medicines with other strategies, comprising immune checkpoint inhibitors (ICIs), chemotherapy, human epidermal growth factor receptor 2 (HER2)-targeted therapies, adoptive cell transfer (ACT), cancer vaccines, and also radiotherapy may have a synergistic anti-tumor impact [24]. This review highlights current knowledge and clinical developments of anti-angiogenesis combination treatment, either alone or in conjunction with other modalities, focusing on last decade in vivo reports.

## Tumor angiogenesis mechanism

Several successive stages throughout tumor angiogenesis may be emphasized. The vessel wall of mature capillaries comprises an endothelial cell lining, a basement membrane, and a layer of cells termed pericytes that partly surround the endothelium [25]. Pericytes share the same basement membrane as endothelial cells and sometimes come into touch with them. Tumor-derived angiogenic agents attach to endothelial cell receptors, initiating the angiogenesis process. VEGF, fibroblast growth factors (FGF), tumour necrosis factor α (TNFα), transforming growth factor (TGF-β), and angiopoietin (Ang) are the most well-known angiogenic cytokines and growth factors [26, 27]. When endothelial cells are encouraged to develop, proteases, heparanase, as well as other digestive enzymes are secreted, which break down the underlying membrane that surrounds the artery [28, 29].

Matrix metalloproteinases (MMPs), a class of metalloendopeptidase produced by tumor cells and supportive cells, allow for the degradation of the basement membrane as well as the extracellular matrix surrounding pre-existing capillaries, typically postcapillary venules [30, 31]. The breakdown of the extracellular matrix also enables the discharge of pro-angiogenic factors out from the matrix. Endothelial cell connections change, cell extensions cross through the gap produced, and the recently created sprout develops towards the source of the stimulation [32]. Endothelial cells enter the matrix and start migrating and proliferating inside the tumor mass. Freshly created endothelial cells arrange into hollow tubes and produce a new basement membrane for vascular stability at this site [33]. The blood flow inside the tumor is formed by freshly shaped fused blood vessels. Significant interactions between cell-associated surface proteins and the extracellular matrix promote the development of the lumen during canalization. Hybrid oligosaccharides galectin-2, platelet endothelial cell adhesion molecule-1 (PECAM-1 or CD31), and VE-cadherin are among the surface proteins discovered in this interaction [34, 35]. Different circumstances, including metabolic and mechanical stressors, hypoxia, and genetic alterations or changed oncogene expression or tumor suppressor genes, may cause an imbalanced shift towards pro-angiogenic factors, while the mechanism behind this is yet unknown.

## Microenvironment role in tumor angiogenesis

Numerous pro-angiogenic agents, such as VEGF, platelet-derived growth factor (PDGF), and FGF are found in the tumor microenvironment. These compounds are produced by cancer cells or tumor-infiltrating lymphocytes or macrophages and can trigger pro-angiogenic signaling pathways, promoting tumor angiogenesis, development, invasion, and metastasis [36]. Furthermore, inflammatory cytokines in the tumor microenvironment have a significant role in tumor angiogenesis. Prior studies have shown that interferon’s (IFNs), TGF-β, and TNF may all have anticancer effects [37]. However, a few investigations have shown that these factors may promote angiogenesis and tumor development. These findings suggest that cytokines have a variety of roles in tumorigenesis as well as development. Numerous interleukin 1 (IL-1) family members stimulate tumor angiogenesis [38]. Through the activity of nuclear factor-kappa B (NF-κB), p38 mitogen-activated protein kinase (MAPK) signaling, and Janus kinase (JAK), IL-1 signaling stimulates angiogenesis by upregulating VEGF as well as angiogenesis-related molecules [39, 40].

**Fig. 2:**
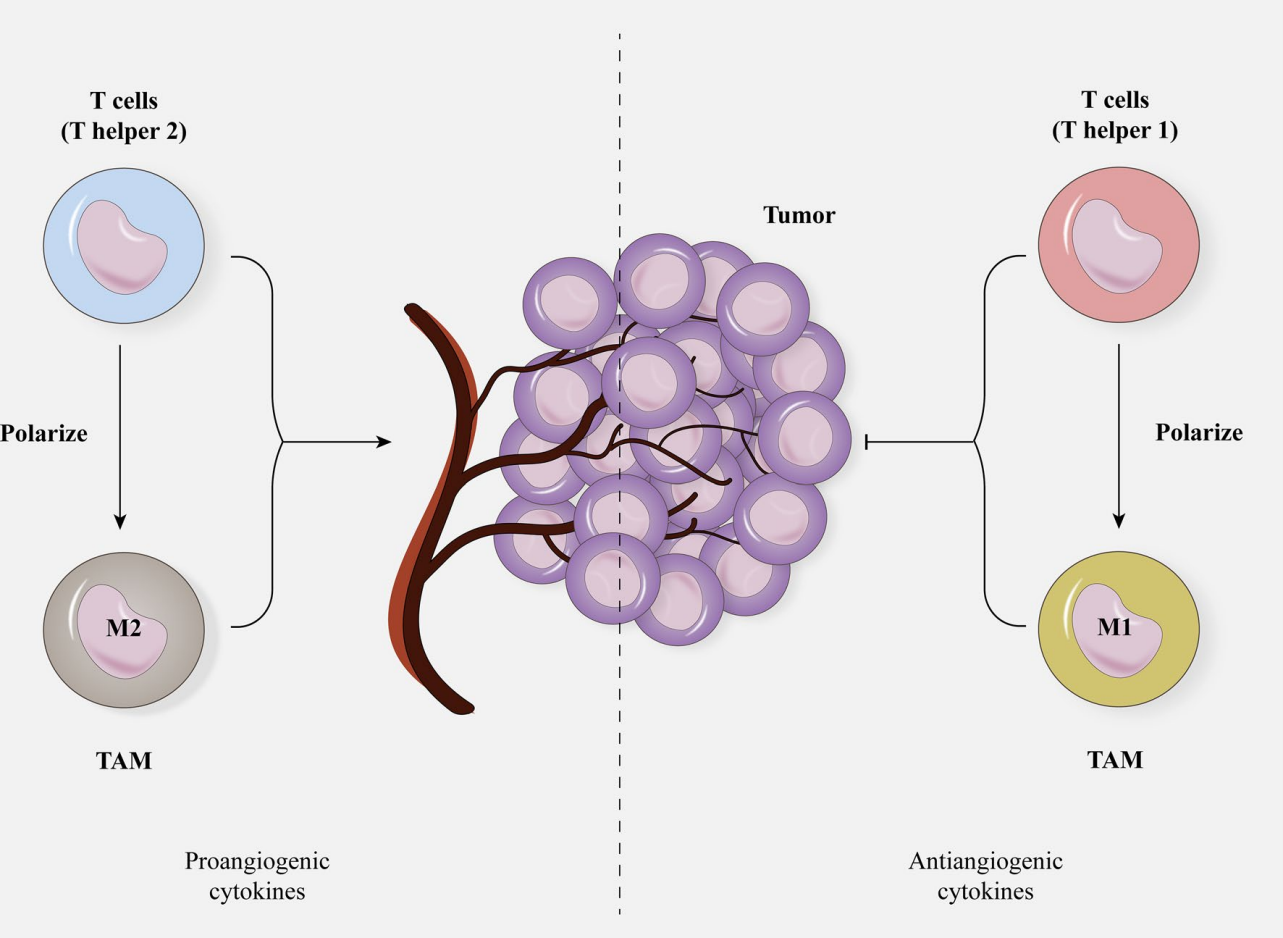
The contrast effects of immune cells found in TME on tumor progress. While TH2 and M2 macrophages convince tumor angiogenesis, TH1 and M1 macrophage suppress tumor angiogenesis by secreting a diversity of cytokines

IL-6, IL-8, and IL-22 may also increase tumor angiogenesis by modulating angiogenic factor expression [41]. A hypoxic microenvironment may also encourage tumor development, invasion, metastasis, immune evasion, and angiogenesis. As a result, co-targeting hypoxic, as well as anti-angiogenic factors, may enhance tumor outcomes. Researchers discovered that co-treatment with hypoxia-inducible factor 1(HIF-1) inhibitors and bevacizumab had a greater anticancer impact than therapy with bevacizumab separately in glioma xenografts [42]. HIF-1 is an upstream regulator of many angiogenic factors that may directly stimulate angiogenic factor transcription to enhance tumor angiogenesis [43]. Furthermore, various hypoxia-induced lncRNAs may enhance tumor angiogenesis by influencing angiogenic factor expression [44]. As angiogenic factors abound in the tumor microenvironment, treating cancer cells with medicines that target several angiogenic agents may result in improved outcomes. Moreover, type 1 T helper (Th1) CD4+ and also CD8 + cells polarize innate immune cells versus tumor regression, for instance by M1 macrophages polarization of tumor-associated macrophages (TAMs) [45]. In contrast, tumor-secreted cytokines largely stimulate a proangiogenic and protumorigenic phenotype of the tumor-associated inflammatory infiltrate. Inducing the type 2 T helper (Th2) CD4 + cells along with regulatory T cells (Tregs) can, in turn, elicit protumoral reactions, comprising M2 polarization of TAMs, culminating proangiogenic microenvironment (Fig. 2) [45]. Recently, Wang et al. showed intra- and inter-tumoral heterogeneities between TAM subpopulations and their functions, with CD86 + TAMs playing a crucial role in tumor progression [46].

## FDA approved anti-angiogenic agents

Upon successful preclinical studies (Table 1), a myriad of clinical trials have been accomplished or are ongoing to determine the safety, feasibility, and efficacy of anti-angiogenic agents therapy in cancer patients alone or in combination with other therapeutic means (Table 2). The present era of anti-angiogenic treatment for cancer research started in 1971 with the publishing of Folkman's creative hypothesis [47], although it would take 33 years for the FDA to authorize the first drug produced as a blocker of angiogenesis. Bevacizumab, a humanized monoclonal antibody targeted against VEGF, was coupled with standard chemotherapy in a randomly selected phase 3 study of first-line therapy of metastatic colorectal cancer (CRC) [48]. When utilized in conjunction with conventional chemotherapy, bevacizumab therapy improved overall survival (OS) in the first-line treatments of advanced non–small-cell lung cancer (NSCLC) [49]. The FDA of the United States has authorized a variety of angiogenesis inhibitors for the treatment of cancer. Most of them are targeted treatments created to target VEGF, its receptor, or other angiogenesis-related molecules. Bevacizumab, axitinib, everolimus, cabozantinib, lenalidomide, lenvatinib, pazopanib, ramucirumab, regorafenib, sorafenib, sunitinib, thalidomide, Ziv-aflibercept and vandetanib are most famous accepted angiogenesis inhibitors, which have been approved for human advanced tumors [50].

**Table 1:** Clinical studies based on angiogenesis blockade therapy alone or in combination with other strategies

Condition (s)	Agent (s)	Participant no	Study phase	Study location	Status	NCT
Ovarian cancer	Apatinib	60	2	China	Unknown	NCT03262545
Colorectal cancer	Regorafenib	59	2	France	Completed	NCT02465502
Thyroid cancer	Axitinib	60	2	USA	Completed	NCT00094055
Non-small-cell lung carcinoma	Axitinib	32	2	USA/Germany	Completed	NCT00094094
Hepatocellular carcinoma	Everolimus Bevacizumab	33	2	Germany	Completed	NCT00775073
Colorectal cancer	Bevacizumab 5-Fluorouracil Oxaliplatin	17	2	USA/Argentina/Italy	Completed	NCT00851045
Solid tumors	JI-101	18	2	USA	Completed	NCT00842335
Non-small-cell lung carcinoma	Paclitaxel Carboplatin CT-322 Bevacizumab	255	2	International	Terminated	NCT00850577
CNS tumor Leukemia Sarcoma	Celecoxib Cyclophosphamide Etoposide Fenofibrate Thalidomide	101	2	USA	Completed	NCT00357500
Colorectal cancer	Cetuximab Ramucirumab Irinotecan hydrochloride	135	2	USA	Active, not recruiting	NCT01079780
Pancreatic cancer	Gemcitabine Axitinib	111	2	International	Completed	NCT00219557
Colorectal cancer	Bevacizumab Capecitabine Levocetirizine	47	2	USA	Completed	NCT01722162
Melanoma	Axitinib	32	2	USA	Completed	NCT00094107
Renal cell carcinoma	Axitinib	52	2	USA/France/Germany	Completed	NCT00076011
Colon cancer	Oxaliplatin Leucovorin 5-Fluorouracil Bevacizumab	70	2	USA/Argentina	Completed	NCT00932438
Colorectal cancer	Bevacizumab Axitinib	187	2	USA	Completed	NCT00460603
Glioblastoma	Topotecan Pazopanib	35	2	USA	Completed	NCT01931098
Breast cancer	Apatinib SBRT	30	2	China	Unknown	NCT03457467
Hepatocellular carcinoma	Brivanib	135	2	International	Completed	NCT00355238
Prostate cancer	Cabozantinib Docetaxel Prednisone	49	2	USA	Completed	NCT01683994
Ovarian cancer	Aflibercept	58	2/3	International	Completed	NCT00327444
Pancreatic cancer	Everolimus	21	2	Germany	Completed	NCT00560963
Melanoma	Sorafenib Bevacizumab	14	2	USA	Completed	NCT00387751
Glioblastoma	Dovitinib	33	2	International	Completed	NCT01753713
Peritoneal cancer Ovarian cancer	Trebananib Paclitaxel	919	3	International	Completed	NCT01204749
Colorectal cancer	Aflibercept Irinotecan 5-Fluorouracil Leucovorin	1226	3	International	Completed	NCT00561470
Pancreatic cancer	Sunitinib	106	4	International	Completed	NCT01525550
Colorectal cancer	Cetuximab Bevacizumab	31	4	France	Terminated	NCT00327093
Pancreatic cancer	Sunitinib	33	2	France	Terminated	NCT01215578
Prostate cancer	Bevacizumab Lenalidomide Docetaxel Prednisone	63	2	USA	Completed	NCT00942578
Colorectal cancer	Bevacizumab Tripleitriuma	50	2	China	Not yet recruiting	NCT04527068
Renal cell carcinoma	Dovitinib Sorafenib	564	3	International	Completed	NCT01223027
Renal cell carcinoma	Pazopanib	80	3	International	Completed	NCT00387764
Endometrial cancer Ovarian cancer Peritoneal cancer Cervical cancer	AL3818 Paclitaxel Liposomal Doxorubicin Topotecan Carboplatin	270	3	USA	Recruiting	NCT02584478
Non-small-cell lung carcinoma	Aflibercept	98	2	International	Completed	NCT00284141
Renal cell carcinoma	Pazopanib	1538	3	International	Completed	NCT01235962

NA

**Table 2:** Combination therapy with anti-angiogenic agents plus other therapeutics in cancer animal models

Cancer	Agent (s)	Study model	Main result	References
Colon cancer	Anti-VEGFR2 plus Anti- PD-1	Mice	Improving the T cell infiltration into, and stimulating local immune activation	[106]
Lung cancer	Anti-VEGF plus Anti-PD-L1	Mice	Induction of T cell activation at higher levels by downregulation of expression of receptor TIM-3 on T cells	[107]
Kidney cancer Breast cancer	Anti-VEGF plus Anti-PD-L1 and Paclitaxel	Mice	Showing the modest anti-tumor effect	[108]
Colon cancer Breast cancer	Apatinib plus Anti-PD-1	Mice	Promoting the efficacy of PD-1 blockade therapy by angiogenesis blockade therapy in VEGFA-overexpressed tumors	[109]
Melanoma	Anti-ANG2 and VEGFA plus Anti-PD-1	Mice	Inducing the robust antitumor effect PD-1 blockade therapy when used in combination with dual Ang2 and VEGFA inhibition	[110]
Melanoma	Anti-VEGFR-1 plus Anti-PD-1 and Anti-CTLA-4	Mice	Reducing tumor growth by promoted M1/M2 and CD8+/FoxP3 + ratios	[111]
Melanoma	Axitinib plus Cancer vaccine	Mice	Attenuation of MDSC and Tregs along with promoting the recruitment of CTLs into tumors	[125]
Glioblastoma	Axitinib plus oHSV-expressing IL-12	Mice	Reduced vascularity, potentiated macrophage infiltration, and huge tumor necrosis	[126]
Prostate cancer Breast cancer Kidney cancer	Sunitinib plus VSV	Mice	Induction of the complete tumor regression in both immunodeficient and immunocompetent animals	[127]
Renal cell carcinoma NSCLC	Sunitinib plus Reovirus	Mice	Reducing tumor growth, improved survival, and reduced MDSCs and Tregs in TME	[128]
Breast cancer	Bevacizumab plus oHSV	Mice	Augmenting the viral distribution and also tumor hypoxia by bevacizumab resulted in tumor cell apoptosis	[129]
Glioma	Bevacizumab plus Oncolytic virus	Mice	Tumor regression and improved survival	[130]
Colon cancer	Lenalidomide plus DCs vaccine	Mice	Marked suppressing tumor growth mainly caused by diminished MDSCs and Tregs, promoted NK cells, and TILs in the spleen	[131]
Lymphoma	Lenalidomide plus IFN-induced DCs vaccine	Mice	Remarkable reduction in tumor growth and lymphoma cell distribution	[132]
Myeloma	Lenalidomide plus DCs vaccine	Mice	Induction of activating antigen-specific cytotoxic T lymphocytes and NK cells, reducing MDSCs and Tregs in the spleen, enhancing TILs population in the spleen, and higher systemic levels of interferon-γ rather than IL-10	[133]
Glioma	Axitinib plus Cyclophosphamide	Mice	Negative modulation of the antitumor actions of metronomic cyclophosphamide by the axitinib (negative effect)	[148]
Ovarian cancer	Bevacizumab plus Paclitaxel and Cisplatin	Mice	Attenuation of tumor progress and metastasis along with improved survival	[149]
Pancreatic cancer	TNP-470 plus Cisplatin	Mice	Showing significant anti-tumor effect by combination therapy, but not monotherapy	[150]
Glioma	TNP-470 plus Temozolomide	Mice	Hindrance of the tumor uptake of temozolomide by pharmacodynamic effects of TNP-470 on the tumor vasculature (negative effect)	[151]
Bladder cancer	TNP-470 plus Cisplatin	Rat	No significant superiority over monotherapy with chemotherapy	[152]
Glioma	Minocycline plus RT and Temozolomide	Rat	Improving the anti-tumor potential of radiotherapy and oral temozolomide leading to prolonged survival by minocycline	[153]
Squamous cell carcinoma	Anginex plus RT	Mice	Prolonged radiation-induced tumor regression	[173]
Squamous cell carcinoma	TNP-470 plus RT	Mice	Tumor regression	[174]
Breast cancer	TNP-470 plus RT	Mice	Potentiating tumor control	[175]
Non-small-cell lung carcinoma	ZD6474 plus RT	Mice	Reduced tumor growth more evidently than monotherapy with radiotherapy	[176]
Non-small-cell lung carcinoma	Honokiol plus RT	Mice	Eliciting synergistic antitumor influences without enhancing toxicity	[178]
Melanoma	Anti-VEGF plus ACT	Mice	Inhibition tumor growth and improved survival	[142]
Non-small-cell lung carcinoma	Endostatin plus cytokine-induced killer cells (CIK) cells	Mice	Promoting the homing of CIK cells and reducing the population of suppressive immune cells in TME	[143]
Neuroblastoma	Bevacizumab plus GD2-redirected CAR T cell	Mice	Increasing the infiltration of CAR T cells to tumor tissue accompanied with improved IFN-γ	[144]
Colon cancer	Regorafenib plus EpCAM redirected CAR-NK-92 cell	Mice	Robust tumor elimination compared with the monotherapy with regorafenib or CAR-NK-92 cells	[145]

*VEGF* vascular endothelial growth factor, *VEGFR2* vascular endothelial growth factor receptor 2, *PD-1* programmed cell death protein 1, PD-L1 programmed death-ligand 1, *CTLA-4* cytotoxic T lymphocyte antigen 4, *oHSV* oncolytic Herpes simplex viruses, *VSV* vesicular stomatitis viruses, *RT* radiotherapy, *EpCAM* epithelial cellular adhesion molecule, *DCs* dendritic cells, *IFN* interferon, *TIM3* T cell immunoglobulin and mucin domain-containing protein 3, *TME* tumor microenvironment, *Tregs* regulatory T cells, *MDSC* myeloid-derived suppressor cells, *Ang2* angiopoietin2, *CTLs* CD8 + cytotoxic T cells, *TILs* tumor-infiltrating lymphocytes, *NK cells* natural killer, *CAR T cells* chimeric antigen receptor T cells

As the first VEGF-targeted agent approved by FDA, bevacizumab, is used since February 2004, for the treatment of patients suffering from metastatic (m) CRC in combination with the standard chemotherapy treatment (as first-line treatment) [51]. In June 2006, it was approved with fluorouracil (5-FU)-based therapy for second-line mCRC. Also, it has been indicated for NSCLC (plus chemotherapy), breast cancer, glioblastoma, ovarian cancer (plus chemotherapy), and also cervical cancer [51]. Another well-known angiogenesis inhibitor, axitinib, has gained approval from FDA for use as a treatment for renal cell carcinoma (RCC) since January 2012 and also has shown promising outcomes in pancreatic cancer (plus gemcitabine) [52, 53]. In March 2009, everolimus was firstly approved for RCC therapy, and after that was indicated for breast cancer patient’s therapy [54]. Moreover, since 2016, it is used for neuroendocrine tumors (NET) of gastrointestinal (GI) or lung origin with unresectable, locally advanced, or metastatic disease [55]. In November 2012, cabozantinib, a small molecule inhibitor of the tyrosine kinases c-Met and VEGFR2, was approved for thyroid cancer [56] and also in April 2016 was accepted as second-line treatment for RCC [57]. Lenalidomide, a 4-amino-glutamyl analogue of thalidomide, is used to treat multiple myeloma (MM) [58] and myelodysplastic syndromes (MDS) [59], and also lenvatinib, which acts as a multiple kinase inhibitor against the VEGFR1, VEGFR2, and VEGFR3 kinases, is applied for the treatment of thyroid cancer [60]. In 2016, lenvatinib was also approved in combination with everolimus for the treatment of advanced RCC [61]. Since 2009, pazopanib, a potent and selective multi-targeted receptor tyrosine kinase inhibitor, is utilized for metastatic RCC and advanced soft tissue sarcomas therapy [62]. Besides, since April 2014, the ramucirumab, a direct VEGFR2 antagonist, is indicated as a single-agent treatment for advanced gastric cancer or gastro-esophageal junction (GEJ) adenocarcinoma after treatment with fluoropyrimidine- or platinum-containing chemotherapy [63]. Further, ramucirumab in combination with docetaxel has gained approval for treatment of metastatic NSCLC [64]. Ramucirumab also is used for mCRC (since 2015) [65] and HCC (since 2019) [66] therapy. Also, regorafenib, an orally-administered inhibitor of multiple kinases, has been indicated for the treatment of patients with advanced HCC who were previously treated with sorafenib [67]. Moreover, sorafenib as another type of kinase inhibitor is used since 2007 for RCC and HCC therapy, and since 2013 for thyroid cancer [68]. Multi-targeted receptor tyrosine kinase inhibitor sunitinib also is applied for gastrointestinal stromal tumor (GIST) and RCC therapy [69]. In addition, since 2006, thalidomide as a type of biological therapy in combination with dexamethasone has been approved for the treatment of newly diagnosed MM patients [70]. Also, Ziv-aflibercept in combination with 5-fluorouracil, leucovorin, irinotecan (FOLFIRI) are used to treat patients with metastatic CRC [71]. Finally, tyrosine kinase inhibitor vandetanib is employed to treat medullary thyroid cancer in adults who are ineligible for surgery [72, 73].

## Resistance to anti-angiogenic therapies

Despite their total tumor growth reduction, therapeutic anti-angiogenic agents were linked to enhanced local invasiveness as well as distant metastasis. These events seem to be significant factors to resistance to anti-angiogenesis treatments. They were originally reported in various preclinical models by Paez-Ribes and coworkers [74]. Based on the literature, anti-angiogenic treatment may increase tumor invasiveness. RCC cells, for example, showed increased proliferation and an invasive character after being treated with bevacizumab [75]. Likewise, glioblastoma cells in mice models were more invasive after VEGF suppression [74]. Sunitinib treatment also has been found to cause vascular alterations such as decreased adherens junction protein expression, reduced basement membrane, pericyte coverage, and increased leakiness [76, 77]. These phenotypic alterations were found in both normal and tumor organ arteries, indicating that they promote tumor cell local intravasation and extravasation, culminating in metastatic colonization [78].

Angiogenesis blockade therapy may lead to vascular regression and resultant intra-tumoral hypoxia. Various investigations have been fulfilled to assess an enhancement in hypoxic areas in primary tumors upon angiogenesis blockade therapy [76, 79]. Further investigation also exposed an attendant augmentation in HIF-1a expression during treatment. HIF-1a and hypoxia are recognized drivers of epithelial-mesenchymal transition (EMT), a process that induced tumor metastasis. Significant improvement in the expression and activities of EMT-related genes (e.g., Twist and Snail) has been observed upon anti-angiogenic treatment and thereby may dampen treatment efficacy [80]. Moreover, loss of the epithelial marker, E-cadherin, and the stimulation of the mesenchymal marker, vimentin, has been evidenced following anti-angiogenic treatment [80]. Hypoxic milieu also largely promotes VEGF expression by the upstream transcription factor HIF-1a [81]. HIF-1, in turn, inspires tumors to achieve more angiogenic and invasive competencies, culminating in metastasis [82]. In fact, hypoxia and EMT bring about increased invasiveness and metastasis of tumors mainly caused by up-regulation of c-Met, Twist, and HIF-1a [83, 84]. Conversely, semaphorin 3A (Sema3A), a well-known endogenous anti-angiogenic molecule, is substantially down-regulated in tumors, ensuring provoked invasiveness and metastasis [85].

Ang-Tie signaling system is a vascular-specific receptor tyrosine kinases (RTK) pathway complicated in modifying the vascular permeability and blood vessel formation and remodeling by potent angiogenic growth factors, Ang-1 and Ang-2 [86]. Molecular analysis has confirmed that activation of the Ang-Tie pathway as a result of the connection between Ang-1 and Tie2 receptor on the M2 subpopulation of monocytes, hematopoietic stem cells (HSCs), and endothelial cells (ECs) of blood and lymphatic vessels elicits maturation or stabilization of blood vessels [80]. Besides, Ang-2 suppresses this pathway, eventually sustaining remodeling or generation of vascular sprouts upon exposure to VEGF [87]. Ang-2 up-regulation has been noticed in multiple types of tumors and is likely involved in resistance versus anti-VEGF therapy [88, 89]. For instance, there is clear evidence signifying that enhanced serum Ang-2 levels are in association with an undesired response to bevacizumab therapy in CRC patients [90]. Studies in lung adenocarcinoma patients revealed that elevated levels of VEGFA and Ang-2 is valued prognostic biomarkers and double targeting of VEGFA and Ang-2 can improve therapeutic outcome [91]. As well, up-regulation and compensatory mechanisms of other growth factors, in particular basic fibroblast growth factor (bFGF), are thought to contribute to the stimulation of the resistance to VEGF targeted therapies. Improved level of the bFGF has strongly been evidenced in the chronic inflammation area, after tissue injury, as well as human cancers bevacizumab [92]. The classical FGF axis can be transduced by RAS/MAPK, PI3K/Akt, Src tyrosine kinase, and STAT pathways, consisting of potent targets for current anti-cancer strategies [93]. Upon bevacizumab treatment in glioblastoma tumor models, Okamoto et al. showed the increased levels of the bFGF and PDGF expression in the endothelial cells, pericytes, and also tumor cells, in turn, caused robust resistance to bevacizumab [94]. Other results indicate that co-targeting of the VEGF and FGF pathways can potentiate tumor cells' sensitivity to bevacizumab, thereby suggesting that the upregulation of the FGF/FGFR autocrine axis plays an indispensable role in eliciting resistance to anti-VEGF/VEGFR therapies [92]. Also, cancer patients with up-regulated bFGF in serum usually show no desired response to sunitinib, indicating the necessity of co-targeting VEGF and bFGF pathways concurrently [95, 96].

Increased metastasis and invasiveness in response to anti-angiogenesis therapy vary according to treatment type, dosage, and schedule. Sunitinib and anti-VEGF antibody monotherapy showed varied effects on mice tumor models, according to Singh et al. reports [77]. While sunitinib therapy increased tumor cell aggressiveness, anti-VEGF antibody treatment did not [77]. Chung et al. also corroborated these findings by comparing the effectiveness of several RTK inhibitors and antibody treatments in mouse models [97]. Though imatinib, sorafenib, or sunitinib increased lung metastasis after 66c14 cell injection, employing an anti-VEGFR2 antibody reduced the development of lung nodules [97]. Overall, reports show that the increased metastasis and invasiveness caused by angiogenesis blockade therapy depend highly on the treatment type.

Anti-angiogenic drug dosage and delivery schedules may also potentially cause resistance. Indeed, short-term and high-dose sunitinib (120 mg/kg per day) therapy before and after intravenous breast tumor cell injection into severe combination immune-deficient animals exhibited the greatest detrimental effects [98]. Sunitinib at high doses accelerated tumor development and facilitated metastasis to the lung and liver, resulting in decreased survival [74, 98]. Although sorafenib had comparable outcomes, sunitinib produced conflicting findings in various trials. High-dose sunitinib therapy before systemic injection of tumor cells enhanced the metastatic potential of lung cancer cells, but not RCC cells. In contrast, low-dose sunitinib (30 and 60 mg/kg per day) had no supportive effect on metastases [78].

## Combination therapy with anti-angiogenic agents

### Anti-angiogenic agents plus ICIs

Recently, scientists have concentrated on the role of immune checkpoint molecules, such as cytotoxic T-lymphocyte antigen-4 (CTLA-4) and programmed cell death protein 1 (PD-1), largely participating in tumor cell escape from immune surveillance as their capacity to obstruct T cell activation [99, 100]. Hence, immune checkpoint inhibitors (ICIs) have been evolved for suppressing these immune checkpoint molecules [101]. FDA-approved ICIs comprise the nivolumab, cemiplimab, and pembrolizumab, atezolizumab, avelumab, durvalumab, and also ipilimumab [102]. Atezolizumab has been approved for use in combination with bevacizumab, paclitaxel, and carboplatin as the first-line treatment of patients with NSCLC [103]. Based on literature, only a subset of PD-L1 positive patients benefits from PD-1/PD-L1 targeted therapies [104]. PD-L1 expression is regulated by various factors, such as inflammatory and oncogenic signaling, leading to the varied significances of PD-L1 positivity. Such alterations in PD-L1 expression lead to the divergent response to PD-1/PD-L1 targeted therapies and may elicit resistance to the PD1/PD-L1 blockade therapies [105].

Recent reports exhibited that combination therapy with anti-angiogenic agents and ICIs could elicit synergistic anti-tumor effects in preclinical models as well as humans (Table 3). Meanwhile, co-administration of anti-PD-1 and anti-VEGFR2 monoclonal antibodies (mAbs) in the Colon-26 adenocarcinoma mice model gave rise to the potent inhibition of tumor growth synergistically without overt toxicity [106]. VEGFR2 blockade therapy negatively regulated tumor neovascularization, as evidenced by the attenuated frequencies of microvessels, whereas PD-1 inhibition exerted no effect on tumor angiogenesis. PD-1 mAbs improved T cell infiltration into tumors and promoted local immune response, as documented via the improvement in various proinflammatory cytokine expressions. Such events signified that concurrent suppression of PD-1 and VEGFR2 might inspire synergistic in vivo anti-tumor influences by dissimilar mechanisms [106]. Further, in a mouse model of small-cell lung cancer (SCLC), co-administration of anti-VEGF and anti-PD-L1 mAbs resulted in a more prominent therapeutic outcome than mono therapy with each agent [107]. Mice that received anti-PD-L1 mAbs alone relapsed after 3 weeks accompanied with a tumor-associated PD-1/ T-cell immunoglobulin domain and mucin domain 3 (TIM-3) double-positive depleted T-cell phenotype. Notably, the depleted T-cell phenotype following anti-PD-L1 therapy was revoked through the addition of anti-VEGF blockade therapy. Analysis revealed that VEGFA expression improves the expression of the inhibitory receptor TIM-3 on T cells, representative of an immunosuppressive action of VEGF in patients with SCLC during PD-1 blockade therapy. Thereby, it seems that VEGFA inhibition may entice T cell activation at higher levels, facilitating T cell-mediated anti-tumor immunity [107]. Similarly, combination therapy with sunitinib and PD-L1 blocked therapy prolonged overall survival (OS) of treated RCC mice models in comparison to mono therapy with either drug [108]. Besides, in the triple-negative breast cancer (TNBC) mice model, PD-L1 blocking was highly effective as an adjuvant monotherapy. However, its co-administration with paclitaxel chemotherapy (with or without VEGF blocked therapy) showed superiority over neoadjuvant therapy [108]. Studies also in VEGFA-overexpressed human tumors and mouse tumor models revealed that apatinib plus PD-1/PD-L1 blockade therapy could alleviate hyperangiogenesis and hypoxia in TME and also alter the immunosuppressive TME into an immunostimulatory microenvironment [109]. Consequently, it was suggested that anti-angiogenesis treatments could potentiate the efficiency of PD-1/PD-L1 blockade therapy in VEGFA-overexpressed tumors [109]. In 2017, Schmittnaegel et al. also noticed that dual Ang-2 and VEGFA inhibition induced antitumor immunity that was promoted by PD-1 blockade therapy in breast cancer, pancreatic neuroendocrine tumor, and melanoma [110]. They showed that Ang-2 and VEGFA blockade by a bispecific antibody (A2V) caused vascular regression, tumor necrosis along with improved antigen presentation by intratumoral phagocytes [110]. The combination therapy also enhanced the presence and activation of interferon-γ (IFNγ)-expressing CD8 + cytotoxic T lymphocytes (CTLs) in tumor tissue, supporting tumor regression [110]. Moreover, anti-VEGFR-1 mAb D16F7 enhanced the antitumor impacts of the anti-CTLA-4 and anti-PD-1 mAbs in B16F10 melanoma cell bearing mice most potently by augmented M1/M2 and CTLs/Tregs ratios, which offer an antitumor and immunostimulating TME [111].

**Table 3:** A summary of clinical trials based on combination therapy with anti-angiogenic agents plus immune checkpoint inhibitors (ICIs) in cancer patients

Cancer	Agent (s)	Main result	References
Renal cell carcinoma	Bevacizumab plus Atezolizumab	Enhancing TILs population in tumor tissue	[197]
Melanoma	Bevacizumab plus Ipilimumab	Improving TILs trafficking, and immune response	[198]
Ovarian cancer	Bevacizumab plus Nivolumab	Inducing significant anti-tumor effect	[113]
Melanoma	Bevacizumab plus Ipilimumab	Improved overall survival	[199]
Ovarian cancer	Bevacizumab plus Atezolizumab and Chemotherapy	No desired effect in newly diagnosed ovarian cancer	[200]
Renal cell carcinoma	Axitinib plus Pembrolizumab	The intervention was tolerable and also resulted in significant objective responsive	[201]
Renal cell carcinoma	Aunitinib or Oazopanib plus Nivolumab	Occurrence of -grade toxicities limiting	[202]
Gastric cancer Renal cell carcinoma	Regorafenib plus Nivolumab	Manageable safety profile with modest anti-tumor effect	[117]
Urothelial carcinoma	Cabozantinib and Nivolumab plus Ipilimumab	Manageable toxicities along with durable responses and prolonged OS	[203]
Renal cell carcinoma	Cabozantinib plus Nivolumab	Improved PFS and OS	[204]
Various tumors	Lenalidomide plus Ipilimumab	Intervention was well-tolerated	[205]
Renal cell carcinoma Endometrial carcinoma	Lenvatinib plus Pembrolizumab	Manageable safety profile with marked objective responsive rate	[206]
Renal cell carcinoma	Lenvatinib plus Pembrolizumab	Manageable safety profile	[207]
Non-small-cell lung carcinoma Gastric/GEJ Hepatocellular carcinoma	Ramucirumab plus Durvalumab	Manageable safety profile with encouraging antitumor activity in patients with high PD-L1 expression	[208]

*gastric/GEJ* gastric/gastro-oesophageal junction adenocarcinoma, *TILs* tumor-infiltrating lymphocytes, *OS* overall survival, *PFS* progression-free survival, *PD-L1* programmed death-ligand 1

Recent clinical trials have also shown that bevacizumab plus atezolizumab could induce synergistic influence on the median OS of patients with RCC [112], and also in combination with nivolumab could elicit modest efficacy in ovarian cancer patients [113]. Also, co-administration of PD-L1 inhibitor avelumab with axitinib resulted in improved objective response rate (ORR) in HCC [114] and also RCC [115] patients, with acceptable safety profile. Also, combination therapy with axitinib and pembrolizumab enhanced median progression-free survival (PFS) in sarcoma patients more evidently than axitinib or pembrolizumab monotherapy. The most common treatment-related unwanted events were autoimmune colitis, pneumothorax, transaminitis, seizures, hemoptysis, and hypertriglyceridemia [116]. Besides, co-administration of regorafenib plus nivolumab resulted in significant antitumor impacts in patients with gastric cancer and CRC [117]. The objective response rate (ORR) was 44% in gastric cancer and 36% in CRC, and also median PFS was 5.6 in gastric cancer and 7.9 months in CRC patients [117]. Moreover, co-administration of nivolumab plus sunitinib or pazopanib showed a significant anti-tumor effect in advanced RCC patients [118, 119]. Conversely, other trials revealed that combined use of regorafenib plus nivolumab [120] and also ramucirumab plus pembrolizumab [121] had no remarkable therapeutic merits in CRC patients [120] and patients with advanced biliary tract cancer (BTC) [121], respectively.

### Anti-angiogenic agents plus cancer vaccines

Therapeutic cancer vaccines ease tumor regression, remove minimal residual disease (MRD), entice durable antitumor memory, and also averts non-specific or adverse events [122, 123]. Till, FDA has approved three cancer vaccines, comprising Bacillus Calmette-Guérin (BCG) lives, sipuleucel-T, and also talimogene laherparepvec (T-VEC) respectively for patients with early-stage bladder cancer, prostate cancer as well as melanoma [124].

In the melanoma mice model, Bose and coworkers found that a treatment regimen comprising a 7-day course of axitinib (0.5 mg/day provided orally) in combination with a vaccine (ovalbumin (OVA) peptide-pulsed syngenic dendritic cells (DCs) adenovirally-engineered to produce anti-angiogenic cytokine IL-12p70) caused remarkable protection versus melanoma progress and prolonged OS when compared to mice receiving each agent alone [125]. These desired outcomes are probably exerted by a decrease in myeloid-derived suppressor cells (MDSC) and Treg frequencies in the tumor concomitant with induction and recruitment of CTLs in TME [125]. Also, addition of the axitinib to oncolytic herpes simplex virus (oHSV) expressing murine IL12 (G47Δ-mIL12) triggered improved OS in both immunodeficient and immunocompetent orthotopic glioblastoma mice models than mice receiving monotherapy [126]. Notably, the addition of the ICI did not promote efficacy in mice models [126]. As well, combination therapy with sunitinib and vesicular stomatitis virus (VSV) brought about the eradication of prostate, breast, and kidney malignant tumors in mice, while monotherapy with VSV or sunitinib did not [127]. Importantly, enhancement in median viral titers by 23-fold following combination therapy indicated that this regimen could potentiate oncolytic virotherapy permitting the recovery of tumor-bearing animals.

In RCC and NSCLC mice model, co-injection of reovirus and sunitinib more potently attenuated tumor burden supporting improved OS, and also reduced the population of immune suppressor cells in tumors compared with monotherapy with reovirus [128]. Thereby, it appears that this regimen can be a rational and effective strategy ready for clinical testing against RCC and NSCLC. Also, Tan and coworkers showed that the bevacizumab improved viral distribution and also tumor hypoxia and promoted the population of apoptotic cells and thus stimulated a synergistic antitumor impact when used in combination with oHSV in TNBC murine models [129]. Combining bevacizumab with OHSV expressing vasculostatin (RAMBO) also demonstrated great anti-tumor capacities in glioma xenografts [130]. Correspondingly, intratumorally administration of RAMBO 1 week after tumor inoculation, and intraperitoneally administration of bevacizumab twice a week reduced migration as well as invasion of glioma cells [130]. Co-treated mice also experienced improved OS and dampened tumor invasion than those treated with bevacizumab alone [130]. In another study, combining tumor antigen-loaded DCs vaccination and anti-angiogenic molecule lenalidomide synergistically potentiated antitumor immunity in the mice colon cancer model, largely provided by suppressing the establishment of immune suppressive cells and also activation of effector cells, such as natural killer (NK) cells [131]. As combination therapy convinced superior polarization of Th1/Th2 ratio in favor of Th1 immune response, it was signified that the applied combination method with DCs and lenalidomide could offer an innovative therapeutic alternative for the amelioration of colon cancer therapy [131]. This regimen similarly caused a robust reduction in tumor growth and malignant cell spread in lymphoma [132] and also myeloma [133] xenografts by similar mechanisms. Further, lenalidomide in combination with a fusion DNA lymphoma vaccine reduced the systemic population of MDSC and Treg in tumor-bearing mice and also led to the decreased tumor burden [134]. In addition, the combination therapy supported the incidence of the higher rates of the antitumor T cells, providing further rationale for clinical application [134].

Currently, a clinical trial was conducted to address the safety and efficacy of combination therapy with sipuleucel-T as a cellular prostate cancer vaccine with bevacizumab in 22 prostate cancer patients [135]. Combination therapy persuaded immune reactions and also alleviated prostate-specific antigen (PSA) in participants with biochemically recurrent prostate cancer [135]. In contrast, co-administration of bevacizumab plus MA950 multi-peptide vaccine adjuvanted with poly-ICLC (polyinosinic-polycytidylic acid stabilized with polylysine and carboxymethylcellulose) did not show superiority over monotherapy with each agent in terms of alteration in OS and PFS in glioblastoma patients [136]. However, a phase II study evaluating the safety and efficacy of bevacizumab in combination with ERC1671, advanced immunotherapy based on freshly extracted tumor cells and lysates, revealed that this regimen could prolong the OS in patients who received ERC1671 plus bevacizumab compared to bevacizumab monotherapy (12 months versus 7.5 months) [137]. Also, there was a tight positive association between the CD4 + T-lymphocyte count and OS in treated patients [137]. Besides, evaluation of the safety, tolerability, and anti-myeloma activity of the PVX-410, a novel tetra-peptide vaccine with 3 of the 4 antigens (XBP1 [2 splice variants] and CD138) with or without lenalidomide was accomplished in MM patients by Nooka et al. [138]. They showed that the PVX-410 vaccine was well tolerated, accompanied by mild injection site reactions and constitutional symptoms. Meanwhile, 5 of 12 patients showed clinical response to combination therapy [138]. The therapeutic values of combination therapy also were verified by an enhancement in frequency tetramer-positive cells as well as IFN-γ cells in the CD3 + CD8 + cell population [138]. Importantly, CRC patients presented complete pathological remission following treatment with bevacizumab, oxaliplatin plus leucovorin and 5-fluorouracil (FOLFOX-4), surgery, and the oncolytic virus Rigvir [139]. In consistence with previous findings, it appears that angiogenesis blockade therapy could promote viral delivery through targeting the TME [139].

### Anti-angiogenic agents plus adoptive cell transfer (ACT)

Adoptive cell therapy (ACT) with using TILs or genetically-modified T cells expressing novel T cell receptors (TCR) or chimeric antigen receptors (CAR) T cells or CAR-NK cells is another plan to convince the immune system to stimulate recognition of the maligned cells and then their eradication [140, 141]. ACT-based immunotherapies can elicit significant tumor regression in animal models and also up to 70% of metastatic melanoma patients. Notwithstanding, tumor vasculature usually obstructs the tumor-specific T cells infiltration, averting anti-tumor immunity. Recent studies delivered proof of the notion that disrupting VEGF/VEGFR-2 signaling could improve the effectiveness of the ACT in tumor model [142]. In the B16 melanoma mice model, co-administration of anti-VEGF mAb to ACT abrogated tumor progress and improve OS [142]. Importantly, anti-VEGF, but not anti-VEGFR-2, antibody considerably augmented infiltration of injected cells into the tumor, suggesting that normalization of tumor vasculature by suppressing VEGF/VEGFR-2 axis could upsurge extravasation of administrated T cells into the tumor [142]. Similarly, anti-angiogenic therapy could also improve the antitumor functions of cytokine-induced killer cells (CIK cells) cells by normalizing tumor vasculature and alleviating the hypoxic TME, as shown in NSCLC xenografts [143]. Meanwhile, Shi et al. evaluated the therapeutic benefits of combination therapy with recombinant human endostatin (rh-endostatin) and CIK cells in NSCLC murine model. They exhibited that rh-endostatin normalized tumor vasculature and attenuated hypoxic regions in the TME [143]. The rh-endostatin markedly potentiated the administrated CIK cells homing and also reduced immune suppressive cells frequency in the tumor tissue. On the other hand, the used regimen instigated a higher level of TILs in tumor tissue [143]. Further, GD2-redirected CAR T cells plus bevacizumab displayed a remarkable anti-tumor effect in an orthotopic xenograft model of human neuroblastoma [144]. Co-administration of bevacizumab or ganglioside GD2-CAR T cells or both by single systemic injection supported higher rates of CAR T cells infiltration into tumor tissue accompanied with improved IFN-γ levels in TME. Additionally, the analysis presented that PD-L1 blockade therapy might augment the efficacy of this regimen [144]. Likewise, epithelial cell adhesion molecule (EpCAM) redirected CAR NK-92 cells injection resulted in CRC cell regression in animal models, which was potentiated when used in combination with regorafenib [145]. These findings delivered a novel plan for the treatment of CRC and also other solid tumors.

### Anti-angiogenic agents plus chemotherapy

Anti-angiogenic agents as noticed can transiently stimulate a functional normalization of the disorganized labyrinth of vessels, sustaining the therapeutic efficacy of coadministered chemotherapeutic agents. Notwithstanding, durable angiogenesis suppression usually fences tumor uptake of chemotherapeutic drugs, and so accomplishment of further studies in this context are urgently required [146]. Correspondingly, designing intermittent treatment schedules is of paramount significance [147].

A study in 9L glioma cell-bearing rats showed that coadministration of axitinib with metronomic cyclophosphamide potently suppressed tumor progress, whereas multiple treatment cycles were needed by monotherapy with metronomic cyclophosphamide to abrogate tumor growth [148]. Importantly, axitinib had no impact on hepatic activation of cyclophosphamide, while it significantly attenuated 9L tumor uptake of cyclophosphamide activated metabolite, 4-hydroxy-cyclophosphamide (4-OH-CPA), by 30–40% [148]. Unfortunately, the abridged tumor infiltration of 4-OH-CPA resulted in a reduction in cyclophosphamide-mediated 9L cell elimination [148]. Such events in turn underlined lacking tumor complete regression by applied combined regimen, reflecting the importance of the optimization of drug scheduling and dosages. In another study, co-administration of the bevacizumab plus cisplatin and paclitaxel concurrently also induced reduced tumor growth as well as improved OS in ovarian cancer xenografts [149]. Also, monotherapy with bevacizumab suppressed ascites formation, accompanied by the partial impact on tumor burden [149]. TNP-470, an angiogenesis inhibitor, plus cisplatin inhibited the liver metastasis of human pancreatic carcinoma [150]. Indeed, liver metastasis percentages reduced from 81.8% in the cisplatin group and 73.3% in the TNP-470 group to 40% in TNP-470 plus cisplatin group. While monotherapy with each agent did not modify tumor growth in vivo, the addition of TNP-470 to cisplatin strikingly reduced tumor growth [150]. Of course, it seems that TNP-470 may entice a decrease in glioma tumor uptake of some chemotherapeutic drugs, such as temozolomide, by affecting the tumor vasculature as a result of its pharmacodynamic effect [151]. As cited, more comprehensive studies are required to define how these combinations can efficiently be utilized. Another study also exhibited that the addition of the TNP-470 to cisplatin chemotherapy reduced the microvascular density of bladder cancer in a murine model [152]. Nonetheless, TNP-470 has no significant influence on the cisplatin impact versus bladder cancer as determined by apoptosis and cell proliferation [152]. Besides, Bow and coworkers demonstrated that local delivery of angiogenesis-inhibitor minocycline could potentiate the anti-tumor efficacy of radiotherapy (RT) and oral temozolomide, as evidenced by enhanced OS in a rodent glioma model [153]. These findings offered further evidence for the idea that angiogenesis inhibitors in combination with conventional therapeutic modalities could promote OS in glioblastoma patients [153].

In 2007, a clinical trial on 25 patients with advanced CRC documented the safety and well-tolerability of combining angiogenesis inhibitor vatalanib, an inhibitor of VEGFR tyrosine kinases, with oxaliplatin/5-FU/leucovorin (FOLFOX4) chemotherapy [154]. Moreover, the addition of the novel anti-angiogenic agent, SU5416, to paclitaxel supported improved PFS accompanied with some mild to modest adverse events (e.g., headache, facial flushing, and fatigue) in patients with head and neck cancer [155]. However, the regimen led to the occurrences of thromboembolic events and prophylactic anticoagulation, suggesting that careful consideration must be taken. Besides, TSU-68 when used plus carboplatin and paclitaxel showed a manageable safety profile in NSCLC patients [156]. Likewise, the addition of the angiogenesis inhibitor ABT-510 (50 mg and 100 mg) as a peptide mimetics of thrombospondin-1 with chemotherapeutic agents (gemcitabine/cisplatin) demonstrated acceptable safety as well as feasibly in patients with NSCLC [157]. Furthermore, combining TNP-470 and paclitaxel was well tolerated with no significant pharmacokinetic interaction between them in NSCLC patients [158]. Further, several clinical trials have verified the efficacy of combination therapy with anti-angiogenic agent and conventional therapy in patients with ovarian cancer [159, 160], CRC [161, 162], NSCLC [163], MCL [164] and also MM [165]. For instance combination therapy with bevacizumab and paclitaxel plus carboplatin prolonged the median OS in participants with platinum-sensitive recurrent ovarian cancer [159]. Also, bevacizumab in combination with low-dose RT and concurrent FOLFIRI induced remarkable objective response (about 39%) in CRC patients [161]. Finally, axitinib combined with cisplatin and gemcitabine [166] and also bevacizumab plus paclitaxel and carboplatin [163] induced significant anti-tumor effect in NSCLC patients, as documented by improved OS and PFS. In addition, the Ziv-aflibercept in combination with 5-fluorouracil, leucovorin, and irinotecan (FOLFIRI) significantly promoted OS in a phase III study of patients with metastatic CRC previously treated with an oxaliplatin-based regimen [167]. However, Ziv-aflibercept in combination with cisplatin and pemetrexed did not significantly affect OS and PFS in patients with previously untreated NSCLC cancer [168].

A list of trials based on combination therapy with angiogenesis inhibitors plus chemotherapy or chemoradiotherapy has been offered (Table 4).

**Table 4:** A summary of clinical trials based on combination therapy with anti-angiogenic agents plus chemotherapy or radiotherapy or chemoradiotherapy in cancer patients

Cancer	Agent (s)	Main result	References
Ovarian cancer	Bevacizumab plus Paclitaxel and Carboplatin	Improved the median overall survival	[159]
Ovarian cancer	Bevacizumab plus Liposomal doxorubicin and Paclitaxel and Topotecan	Improved median overall survival	[160]
Non-small-cell lung carcinoma	Bevacizumab plus Vinorelbine and Gemcitabine and Pemetrexed	No significant effect on median overall survival	[209]
Colorectal cancer	Bevacizumab plus RT and FOLFIRI	The 38.9% of patients experienced a complete response to treatment	[161]
Renal cell carcinoma	Axitinib plus RT	The intervention was well tolerated (3 mg twice daily)	[210]
Colorectal cancer	Bevacizumab plus Oxaliplatin	No effect on disease-free survival or median overall survival	[211]
Non-small-cell lung carcinoma	Axitinib plus Cisplatin and Gemcitabine	Significant anti-tumor activity and with low hemoptysis rate	[166]
Colorectal cancer	Bevacizumab plus RT and FOLFIRI	Significant objective response	[184]
Non-small-cell lung carcinoma	Endostatin plus Chemoradiotherapy	Enhanced progression-free survival and median overall survival without robust toxicity	[212]
Breast cancer	Bevacizumab plus RT	Acceptable safety	[182]
Pancreatic cancer	Bevacizumab plus RT and Erlotinib and Capecitabine	Acceptable safety and tolerability	[183]
Rectal cancer	Bevacizumab plus Capecitabine and RT	Acceptable feasibility	[213]
Non-small-cell lung carcinoma	Bevacizumab plus Paclitaxel and Carboplatin	Significant survival merits with the enhanced treatment-related deaths	[163]
Ovarian cancer	Apatinib plus Etoposide	Promising efficacy along with manageable toxicities	[214]
Mantle cell lymphoma	Lenalidomide plus Rituximab	Durable responses and also manageable safety	[164]
Multiple myeloma	lenalidomide plus Ixazomib and Dexamethasone	Enhanced progression-free survival and median overall survival without robust toxicity	[165]
Rectal cancer	Bevacizumab plus Apecitabine and RT	Significant efficacy along with increased risk of anastomotic leak	[215]
Colorectal cancer	TK/ZK plus Pxaliplatin, 5-FU and Leucovorin	Acceptable safety and feasibility without pharmacokinetic interactions	[154]
Non-small-cell lung carcinoma	Endostatin plus RT	Reduced brain edema without any effect on median overall survival	[216]
Pancreatic cancer	Bevacizumab plus RT	Enhanced acute toxicity	[217]
Rectal cancer	Bevacizumab plus Capecitabine and RT	No effect on progression-free survival and median overall survival	[218]
Colorectal cancer	Bevacizumab plus FOLFIRI and Erlotinib	Improved progression-free survival and median overall survival	[219]
Esophageal cancer	Thalidomide plus RT	Down-regulation of serum levels of VEGF, and also improved treatment outcome	[220]
Non-small-cell lung carcinoma	Sunitinib plus Platinum and Etoposide	No positive anti-tumor effect	[221]
Rectal cancer	Bevacizumab plus Apecitabine and RT	Complete pathological response in 25% of patients concomitant with striking toxicity	[222]
Colorectal cancer	Bevacizumab plus Chemotherapy	Epidermal growth factor-like domain 7 could be described as a biomarkers	[223]
Non-small-cell lung carcinoma	Bevacizumab plus Paclitaxel and Gemcitabine	Improved progression-free survival and median overall survival	[224]
Colorectal cancer	Bevacizumab plus FOLFIRI	Prolonged progression-free survival and median overall survival	[225]
Colorectal cancer	Bevacizumab plus RT	High rate of durable complete responses	[162]
Colorectal cancer	Bevacizumab plus 5-FU and Leucovorin	The regimen was well-tolerated and effective	[226]

*VEGF* vascular endothelial growth factor, *5-FU* fluorouracil, *RT* radiotherapy, *FOLFIRI* folinic acid, fluorouracil, and irinotecan

### Anti-angiogenic agents plus radiotherapy (RT)

RT crucially contributes to the multimodality treatment of cancer. Current evolving in RT have chiefly complicated improvements in dose delivery [169]. Upcoming developments in tumor therapeutics will probably include the combination of RT with targeted therapies. Meanwhile, preliminary results of anti-angiogenic agents in combination with RT have produced encouraging consequences [170]. Further, there are clear proofs that suggest that well-vascularized and perfused tumors mainly exhibit desired response to RT [171, 172].

Studies have shown that the addition of the angiogenesis-inhibitor minocycline to radiotherapy and oral temozolomide could result in prolonged OS in a murine glioma model [153]. Minocycline plus RT enhanced OS by about 140% compared with treatment with RT, while minocycline plus temozolomide improved OS by about 38% compared with monotherapy with temozolomide [153]. Anti-angiogenesis therapy using anginex in combination with RT also supported tumor control in squamous cell carcinoma (SCC) xenografts accompanied by reducing oxygen levels in tumor tissue [173]. Observation showed that the applied regimen modified the amount of functional vasculature in tumors and also augmented radiation-elicited tumor eradication [173]. Likewise, robust hindrance of tumor proliferation was achieved from the addition of the angiogenesis inhibitor TNP-470 to RT in SCC xenografts more evidently than monotherapy with each approach [174]. Also, it was speculated that exclusive investigation of each tumor neovascularization competence can be imperative before deciding the angiogenesis blockade treatment [174]. In contrast, the addition of TNP-470 to RT attenuated the tumor control probability in murine mammary carcinoma [175]. Such unanticipated consequence could be ensured from the partial reserve of reoxygenation by TNP-470, as no remarkable alteration was shown between the RT plus TNP-470 and RT alone under hypoxic conditions [175]. Also, another anti-angiogenic agent, vandetanib (ZD6474) (50 mg/kg), as a potent VEGFR2 tyrosine kinase inhibitor, showed a synergistic effect with RT (3 × 2 Gy) in the NSCLC mice model [176]. Also, vandetanib plus RT strikingly diminished tumor volume by 86% in comparison to the control group in anaplastic thyroid carcinoma (ATC) xenografts [177]. A potent anti-angiogenesis agent, liposomal honokiol, also elicited significant anti-tumor influence by stimulating apoptosis and also suppressing angiogenesis when used plus RT in Lewis lung cancer (LLC) xenografts [178]. Liposomal honokiol, in fact, could ameliorate tumor cell radiosensitivity in vivo, offering that RT plus liposomal honokiol can engender better anti-tumor efficacy in a myriad of tumors, such as lung cancer, SCC, and CRC [178–180].

In 2021, Yang et al. evaluated the safety and efficacy of that combination therapy with axitinib plus RT in advanced HCC patients. They exhibited that the regimen was well tolerated with an axitinib MTD of 3 mg twice daily [181]. Also, the intervention resulted in an ORR of about 66%, comprising 3 complete responses and 3 partial responses among 9 total participants [181]. Besides, the addition of the bevacizumab to adjuvant radiotherapy was associated with the manageable safety profile in breast cancer patients [182]. Meanwhile, grade 3 acute dermatitis was shown in about 10% of patients undergoing combination therapy and 5% of patients undergoing monotherapy with RT without significant modification. Also, pain (18%), fibrosis (8%), and telangiectasia (5%) were the most mutual grade 1–2 side adverse events during 1 years follow-up [182]. Likewise, erlotinib in combination with bevacizumab as well as capecitabine-based definitive chemoradiation (CRT) showed acceptable safety in unresectable pancreatic cancer patients [183]. While 33% of patients showed a grade 3 acute toxicity (including 2 diarrhea, 1 rash), no grade 4 or 5 toxicities were observed during 10 months follow-up. As well 2 of 9 participants showed complete response to intervention [183]. Too, the study of the therapeutic effects of combining RT with FOLFIRI regimen, comprising leucovorin calcium (calcium folinate), 5-fluorouracil, and irinotecan, plus bevacizumab in metastatic CRC also noted objective response in 10/10 patients (3 partial response and 7 complete response) [184]. Similarly, the same regimen caused a partial response in 15/18 or complete response in 4/18 CRC patients, whereas grade 3–4 adverse events toxicity were 2/18 patients [161]. Of course, large-scale trials on this newer therapeutic mean seem justified. Albeit there are some reports which show that combining anti-angiogenic therapy with RT had no therapeutic advantages. For instance, in rectal carcinoma patients, combination therapy with bevacizumab and capecitabine plus RT revealed no merits in terms of improved PFS or OS in the short or long term during a phase 2 clinical trial (NCT01043484) [185].

## Response biomarkers for anti-angiogenic therapy

As a result of some divergences results related to anti-angiogenic agents as well as their modest responses, we must determine and categorize a spectrum of biomarkers, screening the patients of possible responders [186]. Additionally, such biomarkers are urgently required to can monitor disease development and angiogenic actions of tumors following exposure with treatment angiogenesis inhibitors. There are some reports showing that angiogenesis inhibitors could not support therapeutic effect in previously treated metastatic breast cancer [187]. These undesired events are likely related to the secretion of pro-angiogenic factors from resistant malignant tissue [188]. The finding outlines the importance of determining biomarkers to predict the efficacy of VEGF-targeted therapies. Much effort has been spent in this regard and resulted in the finding several biomarkers comprising dynamic measurements (such as variations in systemic blood pressure), circulating markers (such as VEGF serum levels), genotypic markers (such as VEGF polymorphism), blood cells frequencies (such as progenitor cells), tissue markers (such as IFP) and also imaging parameters [such as estimating capillary permeability employing magnetic resonance imaging (MRI)] [189]. Recent studies have revealed that there is a negative correlation between OS with serum lactate dehydrogenase (LDH) and neutrophil levels in CRC patients who received bevacizumab plus standard chemotherapy [190]. Besides, enhanced IL-8 levels were associated with shorter PFS, while low Ang-2 serum levels were related to improved OS in tumor patients undergoing angiogenesis blockade therapy [90]. Circulating endothelial cells (CEC) also has been determined as a robust indicator for the outcome of treatment with bevacizumab. Correspondingly, patients with less than 65 CEC/4 mL blood at baseline mainly experienced prolonged OS and PFS [191]. Besides, patients with IL-6 G-174C and P53 codon 72, MMP9 C-1562T, and CXCR-1 G + 2607C polymorphism may exhibit the favored response to anti-angiogenic agents [191]. On the other hand, greater intra-tumoral expression of VEGFR-3 may predict better response, while overexpression of VEGFR1 mainly indicates poor survival [192]. Other studies in RCC patients upon treatment with sorafenib also revealed that high baseline levels of VEGF were related to poor prognosis [193], while serum levels of circulating neutrophil gelatinase-associated lipocalin (NGAL) and VEGF were powerfully supported prolonged PFS in RCC patients receiving sunitinib [194].

## Conclusion and prospect

**Fig. 3:**
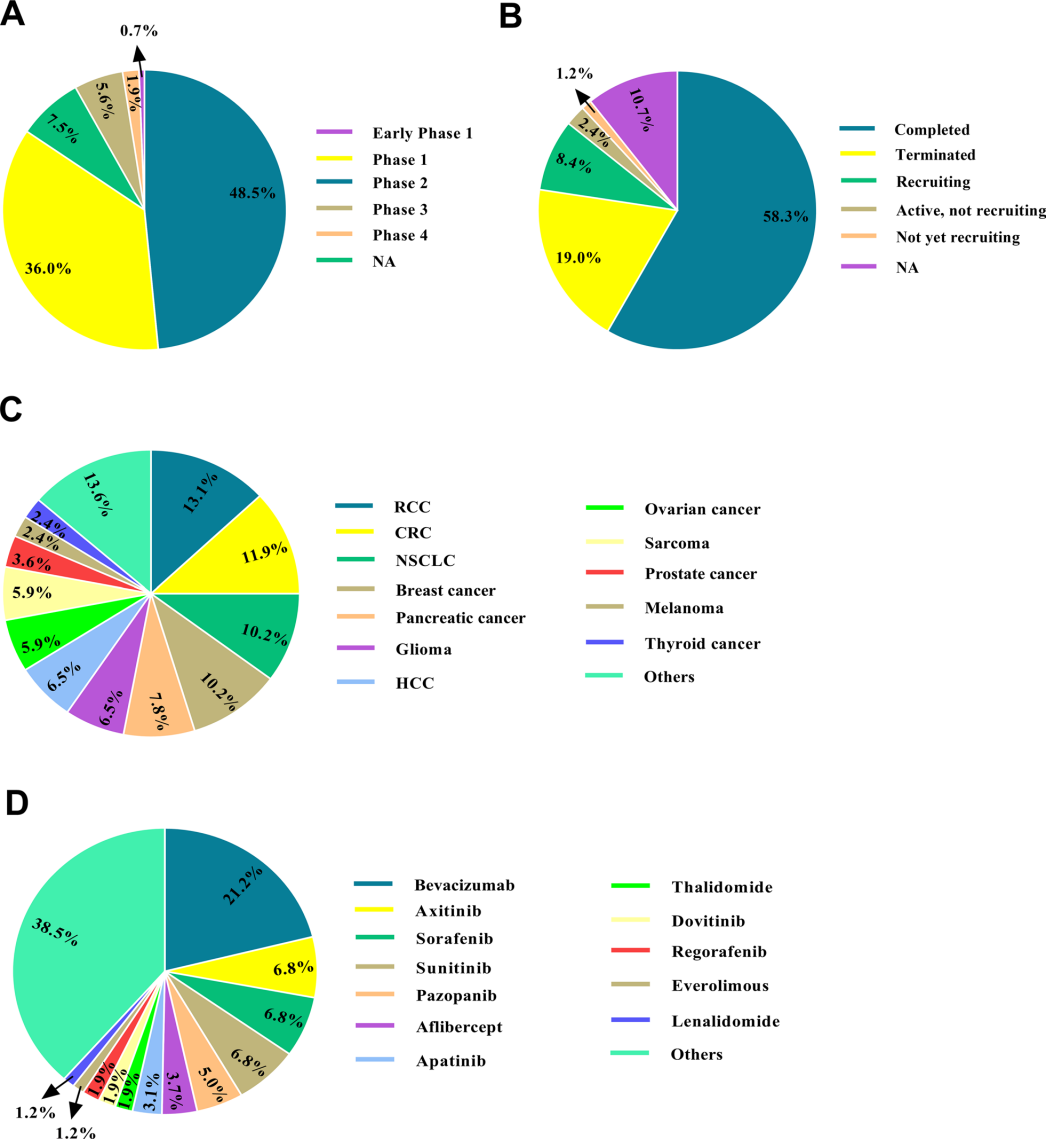
Clinical trials based on cancer therapy by anti-angiogenic agents registered in ClinicalTrials.gov (October 2021). The schematic exemplifies clinical trials utilizing anti-angiogenic agents depending on the study status (**A**), study phase (**B**), study location (**C**), and condition (**D**) in cancer patients

In contrast to the classical hypothesis of vascular regression, the central aim of conventional anti-angiogenic treatments is tumor vascular normalization and maturity. This event, in turn, offered enhanced tumor access to chemotherapeutic drugs and underlays more efficient cancer immunotherapy. As cited, survival benefits of angiogenesis blockade therapy are compromised by cancer resistance to theses agent, and thereby provoke interest in evolving more effective means to combine anti-angiogenic drugs with other conventional therapeutics. To date, a large number of clinical trials have evaluated the safety and therapeutic merits of angiogenesis blockade therapy alone or in combination with other modalities in cancer panties (Fig. 3). Although combination therapy regimen mainly caused significant efficacy in cancer patients, intervention-related toxicities hurdle their application in clinic. For instance, bevacizumab therapy could sustain ischemic heart disease. Indeed, CRC patients receiving bevacizumab may experience considerably augmented possibility of cardiac ischemia [195]. In addition, it has been proved that combination therapy with angiogenesis inhibitors and chemotherapeutic agents may attenuate antitumor effects of chemotherapy. Hence, further rigorous investigations are warranted to circumvent the cited problems. Moreover, determining the suitable dose and sequence is of paramount importance to optimize the effectiveness, toxicity, and tolerability of the combination therapy. Thanks to the involvement of a myriad of cytokines and growth factors and the resultant interplay and compensation among them, co-targeting various growth factors is urgently required. The recognition and potent suppression of downstream kinases and strategic signaling biomolecules where several angiogenic pathways converge may defeat current difficulties motivated via the variety of angiogenic ligands and receptors and should be the emphasis of upcoming investigations. For instance, dual EGFR inhibition (erlotinib and cetuximab) combined with bevacizumab is a safe and well-tolerated combination, demonstrating antitumor activity in patients with solid tumors [196]. BQ13esides, continued treatment with conventional anti-angiogenic agents is related to toxicity and drug resistance. These conditions offer a robust justification for novel plans to improve the efficacy of mAbs targeting tumor vasculature, such as antibody–drug conjugates (ADCs) and peptide-drug conjugates (PDCs), offering a new avenue to exert anti-angiogenic effects on cancerous cells.

## Data Availability

Not applicable.
